# Comparative proteomic analysis provides insights into the complex responses to *Pseudoperonospora cubensis* infection of cucumber (*Cucumis sativus* L.)

**DOI:** 10.1038/s41598-019-45111-4

**Published:** 2019-07-01

**Authors:** Peng Zhang, Yuqiang Zhu, Xiujun Luo, Shengjun Zhou

**Affiliations:** 1 Institute of Vegetable, Zhejiang Academy of Agriculture Sciences, Hangzhou, China; 2 College of Life and Environmental Science, Hangzhou Normal University, Hangzhou, 310036 China

**Keywords:** Proteomics, Plant breeding

## Abstract

Cucumber (*Cucumis sativus* L.) is an important crop distributed in many countries. Downy mildew (DM) caused by the obligate oomycete *Pseudoperonospora cubensis* is especially destructive in cucumber production. So far, few studies on the changes in proteomes during the *P*. *cubensis* infection have been performed. In the present study, the proteomes of DM-resistant variety ‘ZJ’ and DM-susceptible variety ‘SDG’ under the *P*. *cubensis* infection were investigated. In total, 6400 peptides were identified, 5629 of which were quantified. KEGG analysis showed that a number of metabolic pathways were significantly altered under *P*. *cubensis* infection, such as terpenoid backbone biosynthesis, and selenocompound metabolism in ZJ, and starch and sucrose metabolism in SDG. For terpenoid backbone synthesis, 1-deoxy-D-xylulose-5-phosphate synthase, 1-deoxy-D-xylulose 5-phosphate reductoisomerase, 2-C-methyl-D-erythritol 2,4-cyclodiphosphate synthase, 4-hydroxy-3-methylbut-2-en-1-yl diphosphate synthase, and geranylgeranyl pyrophosphate synthase were significantly accumulated in ZJ rather than in SDG, suggesting that pathogen-induced terpenoids accumulation might play an important role in the resistance against *P*. *cubensis* infection. Furthermore, a number of pathogenesis-related proteins, such as endochitinases, peroxidases, PR proteins and heat shock proteins were identified as DAPs, suggesting that DM resistance was controlled by a complex network. Our data allowed us to identify and screen more potential proteins related to the DM resistance.

## Introduction

Cucumber (*Cucumis sativus* L.) is a poplar vegetable crop cultivated all over the world, and its yield and quality is susceptible to various pathogen infections^1,2^. Downy mildew (DM), caused by the oomycete *Pseudoperonospora cubensis*, is the major destructive disease of cucumber^3^. Uncontrolled application of fungicides, although providing some level of disease control, causes environmental pollution and drug resistance^4^. Identification and isolation of genes and proteins associated with DM responses was proven to be an efficient way to build up the host resistance in cucumber cultivars^5^. However, the regulation mechanism underlying the responses to *P*. *cubensis* infection has been reported, to date.

In 2004, two plant eR genes, *At1* and *At2*, encoding the photorespiratory peroxisomal enzymes were reported to be involved in the resistance to the *P*. *cubensis* infection^6^. An involvement of potential NBS-encoding resistance gene, *CsRGA23*, in the defense responses to *P*. *cubensis* has been reported by Wan’s group^7^. A cucumber *Heat Shock Protein* (*HSP45*.*9*) gene was significantly induced by the *P*. *cubensis* infection, suggesting a role of *CsHSP45*.*9* in the DM resistance of cucumber^8^. *CsERF004* in the susceptible cultivar induced the expression of *CsPR1* and *CsPR4* and the contents of salicylic acid and ethylene, indicating that *CsERF004* conferred the resistance to *P*. *cubensis*^9^. In addition, several QTLs for the DM resistance in cucumber also have been recently reported^10^. For example, DM-resistant cucumber inbred ‘WI7120’ was used for QTL mapping, resulting four QTLs, including two major effect QTLs, one additive effect QTL, and one minor effect QTL^11^. Using an F2 population for DM-resistant cucumber variety, five QTLs were identified, among which *dm2*.*2* displayed the largest effect on the DM resistance^12^.

Many studies on the proteomic variations in cucumber during various treatment conditions have been done. A comparative proteomic analysis revealed 63 differential accumulated proteins (DAPs), giving new insights into salicylic acid responses in cucumber seedlings^13^. ITRQ-based quantitative proteomics approach provided integrated insights into salinity responsive mechanism in cucumber phloem sap samples^14^. Another comparative proteomic analysis identified 221 DAPs involving in 30 metabolic pathways, revealing the positive role of exogenous spermidine in photosynthesis efficiency and salinity tolerance of cucumber plants^15^. Using MALDI-TOF/TOF MS method, 62 DAPs in the roots of cucumber under NaCl stress were identified^16^. Taking advantage of cucumber waterlogging tolerant variety ‘Zaoer-N’ and sensitive variety ‘Pepino’, several key proteins involved in adventitious root emergence under waterlogging stresses were identified by iTRAQ-based quantitative proteomics approach^17^. Recently, ten up- and four down-regulated proteins were identified in ABA/H_2_O_2_ induced adventitious roots in cucumber under drought stress^18^.

Recently, a novel ‘tandem mass tags’ (TMTs) strategy was developed for large scale protein quantification^19^. So far, several cucumber transcriptomes responsive to the *P*. *cubensis* infection have been published by different research groups. A number of genes associated with the resistance to *P*. *cubensis* infection were identified using suppression subtractive hybridization in cucumber^5^. Expression profiling throughout a time course of the host response to *P*. *cubensis* was performed using whole transcriptome sequencing^20^. Additionally, a comprehensive transcriptomic analysis of resistant and susceptible cucumber seedlings during *P*. *cubensis* infection created co-expression modules containing genes associated with earlier response to the pathogen^21^. However, very few proteomic data on cucumber under the *P*. *cubensis* infection have been reported. Our data allowed us to identify and screen more potential proteins related to the DM resistance.

## Materials and Methods

### Cucumber materials and sampling

A DM-resistant cultivated cucumber variety ‘ZJ’ and a DM-susceptible cultivated variety ‘SDG’ were used. Cucumber seedlings were planted in a growth chamber with photoperiod of 12 h light/12 h dark, relative humidity of 60%, and light intensity of 120 μmol m^−2^ s^−1^. A solution containing *P*. *cubensis* sporangia (2 × 10^6^ sporangia/mL), 5 mM glucose, and 2.5 mM KH_2_PO_4_ was prepared as the inoculant. The same solution without pathogen was used as negative control. For inoculation, the third leaves of seedlings at the three-leaf stage were sprayed with *P*. *cubensis* sporangia or control solution. All inoculated seedlings (four groups × three replicates) were kept in the same condition and separately covered with plastic films. In total, 10 seedlings were contained in each group. The third leaves of seedlings at the three-leaf stage were harvested for protein isolation. Plant samples for each group were harvested at 48 h post inoculation and cleaned with deionized H_2_O, and then were immediately frozen in liquid N_2_ until use. To get a comprehensive understanding of the proteins involved in the DM resistance, four sample groups, including DM-resistant variety treated with *P*. *cubensis* sporangia (ZJT), DM-resistant variety treated with control solution (ZJC), DM-susceptible variety treated with *P*. *cubensis* sporangia (SDGT), and DM-susceptible variety treated with control solution (SDGC), were used for comparative proteomic analysis.

### Protein extraction

About 500 mg plant samples for each replicate were grinded by liquid N_2_ into cell powder. Four volumes of pre-cooled lysis buffer containing urea (8 M), Triton-100 (1%), dithiothreitol (10 mM), and protease inhibitor (1%) was added, followed by sonication five times using an ultrasonic processor (Scientz, Ninbo, China) on ice. The remaining debris was discarded by centrifugation at 12,000 × g at 4 °C for 10 min. At last, the peptide samples were precipitated with 20% of cold TCA buffer for 3 h at −20 °C. The supernatant was removed by 12,000 × g centrifugation at 4 °C for 15 min. The remaining precipitate was cleaned with pre-cooled acetone for three times and was re-dissolved in urea solution (8 M). The proteins were quantified by a GE Healthcare 2-D Quant kit (Beijing, China).

### Trypsin digestion

Sample solution was reduced with dithiothreitol (5 mM) for 30 min and alkylated with iodoacetamide (11 mM) for 15 min at 25°C in darkness. Then, TEAB (100 mM) was added to dilute the protein solution to concentration of 2 M. For trypsin digestion, trypsin was added at trypsin/protein mass ratio of 1:50 for the first overnight digestion and at mass ratio of 1:100 for the second 4 h digestion.

### TMT/iTRAQ labeling

Tryptic peptide sample was desalted by a Strata X-C18 SPE column (Phenomenex, Torrance, USA). Then, the dried peptide samples were re-constituted in TEAB solution (0.5 M) and manipulated using TMT/TRAQ kit (Thermo-Scientific, Shanghai, China) according to the its protocol. One unit of TMT reagent was dissolved and reconstituted in acetonitrile. The resulting solution was incubated for 2 h at 25 °C. At last, the result peptides were pooled, desalted and dried.

### Fractionation and LC-MS/MS analysis

The column was wetted with 0.1% formic acid in acetonitrile and conditioned with ddH_2_O. The labeled samples were loaded onto an Agilent 300Extend C18 column (5 μm particles, 4.6 mm ID, 250 mm length, Santa Clara, CA, USA) for high pH reverse-phase HPLC. A solution, containing 0.1% formic acid in 98% acetonitrile, at a flow rate of 400 nL/min was used for the EASY-nLC 1000 UPLC system (Thermo Scientific, Beijing, China). The gradient was increasing from 6% to 25% in 26 min, from 25% to 35% in 8 min, climbing to 80% in 3 min, and holding at 80% for 5 min.

The electrospray voltage was set at 2000 V. The range of m/z full scan was set from 350 to 1800. Intact peptides were identified in the Orbitrap at a high resolution of 70,000 and the fragments were identified in the Orbitrap at a low resolution of 17,500. Automatic gain control was set at 5E4. The first mass was fixed at 100 m/z. The MS proteomics data were deposited to the Proteome EXchange Consortium via the PRIDE partner repository with the identifier PXD010844.

### Quantification of identified proteins

The ratios of the TMT reporter ion intensities in MS/MS spectra (*m/z* 126–131) were applied to compute changes in each protein between different samples. Data of each protein was mean-normalized to center the distribution of quantitative values. Then, protein quantitation was computed as the median ratio of corresponding peptides for a unique protein. Two-sided *t*-tests were applied to analyze the expression differences of proteins from two sample groups. A significance level at 0.05 was used for statistical testing.

### Searching and annotation of protein

The MS/MS data were searched against Cucumber Genome with an integrated MaxQuant engine (v.1.5.2.8) with default parameters. The mass tolerance of precursor ions was allowing up to 20 ppm in the first round searching and up to 5 ppm in the main searching. The other parameters were set according to the previous published work^22^.

Gene Ontology (GO) annotation of each protein was derived from the UniProt-GOA database. Firstly, all identified protein were uploaded onto UniProt database to get their IDs and then were searched against the GO database. Domain functional description of identified protein were annotated by the InterPro database. For Kyoto Encyclopedia of Genes and Genomes (KEGG) annotation, KEGG online service tool ‘KAAS’ was used to annotate the identified proteins. Then, the annotated proteins were searched against the KEGG database using another online tool ‘KEGG mapper’.

The cells of eukaryotic organisms are elaborately subdivided into functionally distinct membrane bound compartments. In our study, Wolfpsort (http://wolfpsort.seq.cbrc.jp/), a subcellular localization predication software, was used to predict subcellular localization.

For enrichment analysis, a two-tailed Fisher’s exact test was applied to analyze the GO, KEGG and domain enrichments of the differential abundance proteins (DAPs) against all the identified proteins. Correction for multiple hypothesis testing was performed using standard FDR control method^23^.

### Validation of DAPs by parallel reaction monitoring (PRM)

To validate the DAPs under the *P*. *cubensis* infection, the changes in several key proteins were identified by an acquired MS/MS spectrum. Three independent biological replicates were used in this experiment. The peptides were treated with trypsin and the tryptic peptides were dissolved in 0.1% FA solution. The resulting peptide samples were loaded onto a 150 mm length, 75 μm, reversed-phase analytical column (Thermo, Shanghai, China). The gradient was comprised of an increase from 7% to 25% solvent B (0.1% formic acid in 98% acetonitrile) over 38 min, 25% to 36% in 14 min and climbing to 80% in 4 min then holding at 80% for the last 4 min, all at a constant flow rate of 700 nL/min on an EASY-nLC 1000 UPLC system.

The resulting MS data were processed using Skyline (v.3.6). Peptide settings: enzyme was set as Trypsin [KR/P], Max missed cleavage set as 2. The peptide length was set as 8–25, Variable modification was set as Carbamidomethyl on Cys and oxidation on Met, and max variable modifications was set as 3. Transition settings: precursor charges were set as 2, 3, ion charges were set as 1, 2, ion types were set as b, y, p. The product ions were set as from ion 3 to last ion, the ion match tolerance was set as 0.02 Da.

### Statistical analysis method

Significant analysis was performed using a one-way analysis of variance with a Tukey’s test. All values are showed using the averages of three replicates, and data are showed as the mean with the standard deviation.

## Results

### Overview of MS data

The proteomes of two cucumber varieties under the control and *P*. *cubensis* infection conditions were analyzed in our study. Correlation coefficients of 12 samples (three replicates × four groups) showed a well repeatability of our data (Fig. S1a). Quality validation showed that distribution of mass errors was lower than 0.02 Da and lengths of peptides varied from 7 to 20 amino acid residues, indicating a good quality of our sample preparation (Fig. S1b,c).

### Impacts of the *P*. *cubensis* infection on proteome levels in cucumber

**Figure 1:**
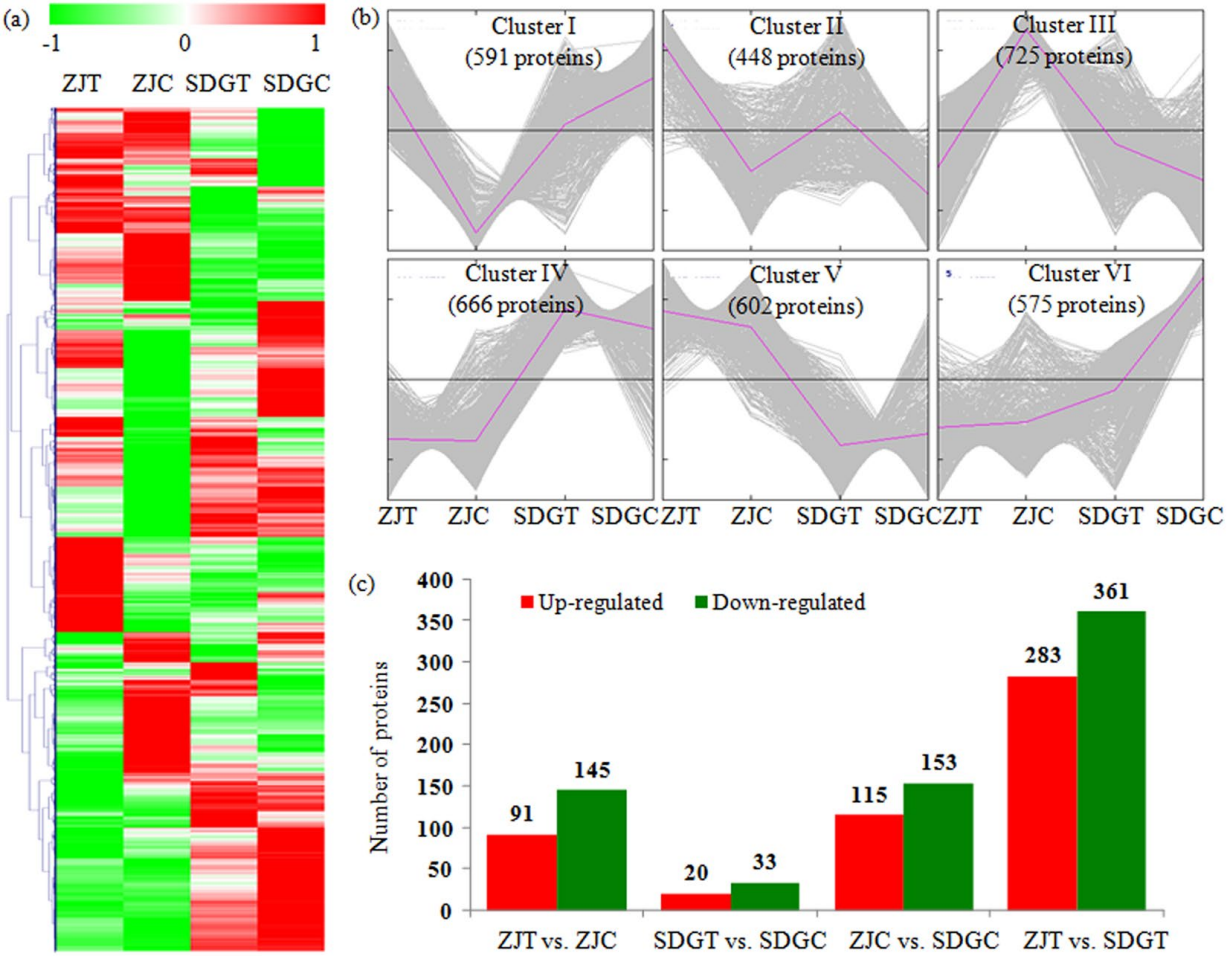
Impacts of *P*. *cubensis* infection on proteome levels in cucumber. (**a**) Expression profiles of the DAPs response to *P*. *cubensis* infection. (**b**) All DAPs were analyzed and clustered into six major Clusters by K-means method. (**c**) The numbers of up- and down-regulated proteins in various comparisons, including the ZJT vs. ZJC, SDGT vs. SDGC, ZJC vs. SDGC, and ZJT vs. SDGT comparisons.

To compare the DAPs between different sample groups, expression profiles of the quantified proteins were showed in a heatmap (Fig. 1a). To reflect the changing trends among different sample groups, all of the quantified proteins were assigned into six clusters using MeV software by K-means method. The proteins in the cluster I showed the highest levels in the ZJT and SDGC samples; the proteins in the cluster II highly accumulated in the ZJT sample; the proteins in the cluster III predominantly accumulated in the ZJC sample; the proteins in the cluster IV showed highest levels in SDG; the proteins in the cluster V showed the highest levels in ZJ; and the proteins in the cluster VI predominantly accumulated in the SDGC sample (Fig. 1b).

In the ZJT vs. ZJC comparison, 236 DAPs, including 145 induced and 91 reduced proteins, were identified; in the SDGT vs. SDGC comparison, 53 DAPs, including 33 induced and 20 reduced proteins, were identified; in the ZJC vs. SDGC comparison, 268 DAPs, including 153 induced and 115 reduced proteins, were identified; and in the ZJT vs. SDGT comparison, 644 DAPs, including 361 induced and 283 reduced proteins, were identified (Fig. 1c).

Among the DAPs between ZJC and ZJT, the top five up-regulated proteins were a thaumatin-like protein (Csa5G155450.1), a pathogen-related protein (Csa6G338110.1), an acidic endochitinase (Csa5G139760.1), a pathogenesis-related protein P2 (Csa2G010390.1), and an 1-aminocyclopropane-1-carboxylate oxidase (Csa3G135720.1), and the top five down-regulated proteins were a proto-chlorophyllide reductase (Csa4G638340.1), a 10 kDa chaperonin 1 (Csa6G080390.1), an acetolactate synthase small subunit 2 (Csa1G652220.1), a tetrapyrrole-binding protein (Csa6G042370.1), and an RNA-binding protein CP29B (Csa3G740070.1) (Table S2). Among the DAPs between SDGC and SDGT, the top five up-regulated proteins were a pathogen-related protein (Csa6G338110.1), a phenylalanine ammonia-lyase (Csa6G446290.1), an acidic endochitinase (Csa5G139760.1), a glutathione S-transferase (Csa1G024860.1), and a peroxidase 73 (Csa6G495000.1), and the top five down-regulated proteins were a small heat shock protein (Csa5G138480.1), a small heat shock protein (Csa3G020080.1), a 17.9 kDa class II heat shock protein (Csa4G646340.1), a BEL1-like homeodomain protein (Csa3G782620.3), and a heat shock 70 kDa protein (Csa2G070310.1) (Table S3).

### Protein annotation and classification

In our study, 6400 proteins were identified, 5629 of which were quantified. The detail information, including protein IDs, GO, KEGG and domain categories, subcellular localizations, and functional enrichments, of all identified proteins were listed Table S1.

**Figure 2:**
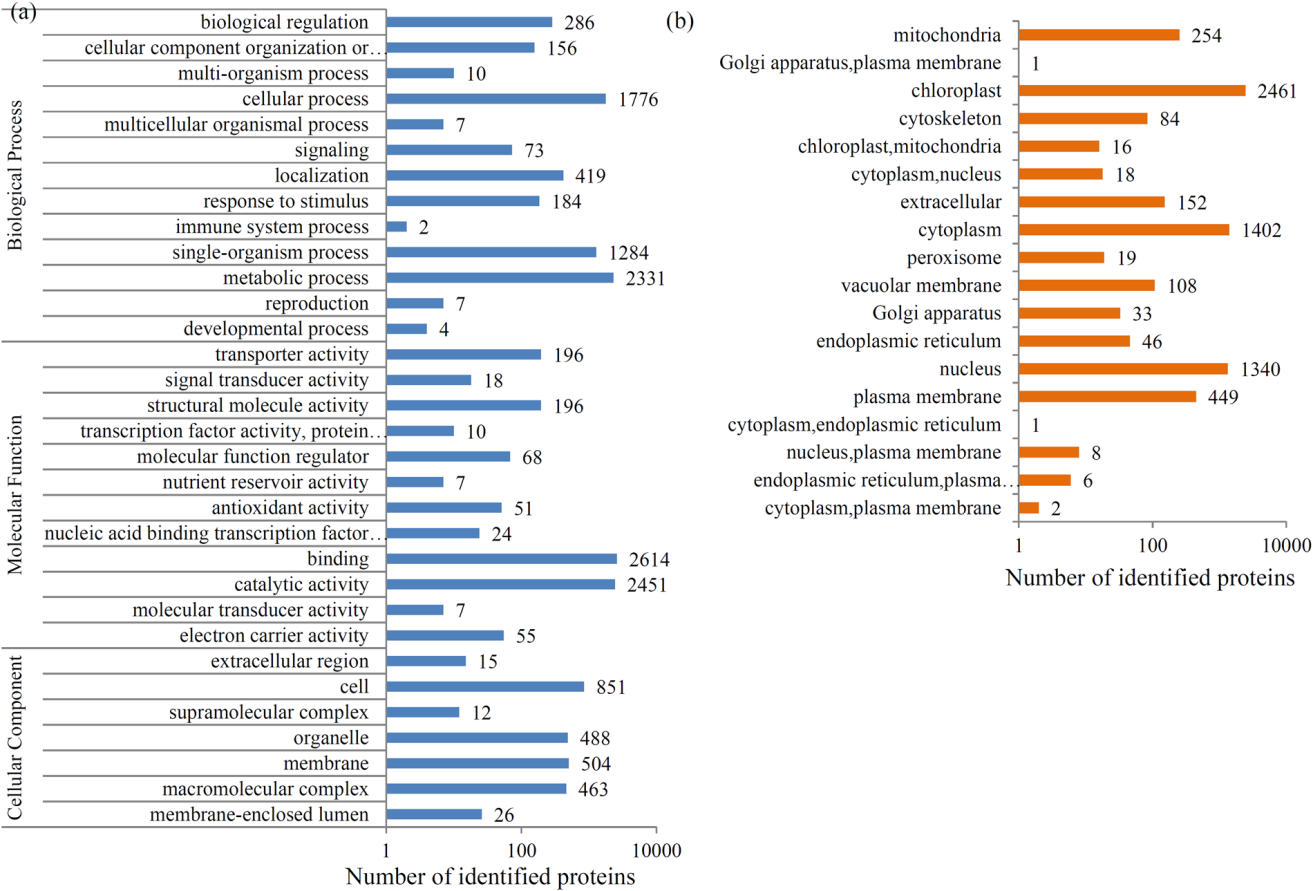
Protein annotation and classification. (**a**) All identified proteins were classified by GO terms based on their cellular component, molecular function, and biological process. (**b**) Subcellular localization of all the identified proteins.

Most of the identified proteins could be assigned into various functional GO terms. For the biological process category, ‘metabolic processes’ (2331 proteins), ‘cellular processes’ (1776 proteins) and ‘single-organism process’ (1284 proteins) were the dominant terms; for the cellular component category, the largest terms were ‘cell’ (851 proteins), ‘membrane’ (504 proteins) and ‘organelle’ (488 proteins); and for the molecular function category, most proteins were related to ‘binding’ (2614 proteins), ‘catalytic activity’ (2451 proteins), “binding” (5,168 unigenes), ‘transporter activity’ (196 proteins) and ‘structural molecule activity’ (196 proteins) (Fig. 2a). According to their subcellular localizations, most identified proteins were grouped into 18 subcellular component categories, such as 2461 chloroplast-, 1402 cytoplasm-, 1340 nucleus-, and 499 plasma membrane-localized proteins (Fig. 2b).

### Enrichment analysis of the DAPs in both ZJ and SDG under the *P*. *cubensis* infection

**Figure 3:**
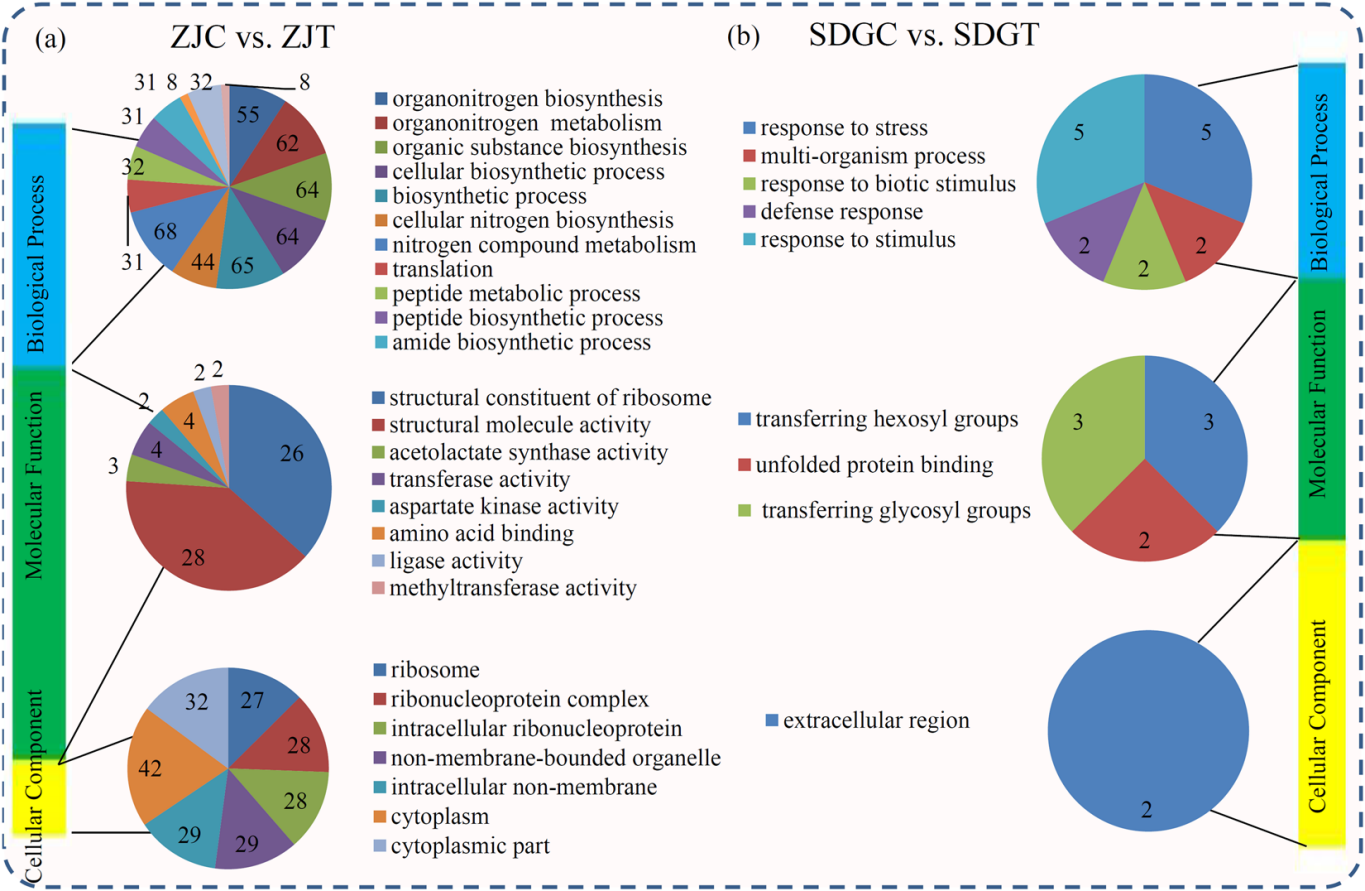
GO enrichment analysis of the DAPs in different comparisons. GO terms distribution of the DAPs in the ZJT vs. ZJC comparison (**a**) and the SDGT vs. SDGC comparison (**b**). Different color blocks represent different terms, including cellular component, molecular function, and biological process. Number of the DAPs in each second level term was showed in a pie chart.

For the DAPs in ZJ, the significantly enriched biological process GO terms were related to ‘nitrogen compound metabolism’ (68 DAPs), ‘biosynthetic process’ (65 DAPs), ‘cellular biosynthetic process’ (64 DAPs), and ‘organic substance biosynthesis’ (64 DAPs); the majority of the DAPs categorized in the biological process associated GO terms were grouped into the ‘structural molecule activity’ (28 DAPs) and ‘structural constituent of ribosome’ (26 DAPs); the significantly enriched molecular function GO terns were ‘cytoplasm’ (42 DAPs), ‘cytoplasmic part’ (32 DAPs), ‘intracellular non-membrane’ (29 DAPs) and ‘non-membrane-bounded organelle’(29 DAPs) (Fig. 3a and Table S4). For the DAPs in SDG, the most enriched biological process GO terms were ‘response to stress’ (5 DAPs), ‘response to stimulus’ (5 DAPs), ‘multi-organism’ (2 DAPs), ‘response to biotic stimulus’ (2 DAPs), and ‘defense response’ (2 DAPs); the largest molecular function GO terms were ‘transferring hexosyl groups’ (3 DAPs), ‘transferring glycosyl groups’ (3 DAPs), and ‘unfolded protein binding’ (2 DAPs); the significantly enriched component function GO terms was ‘extracellular region’(2 DAPs) (Fig. 3b and Table S5).

**Figure 4:**
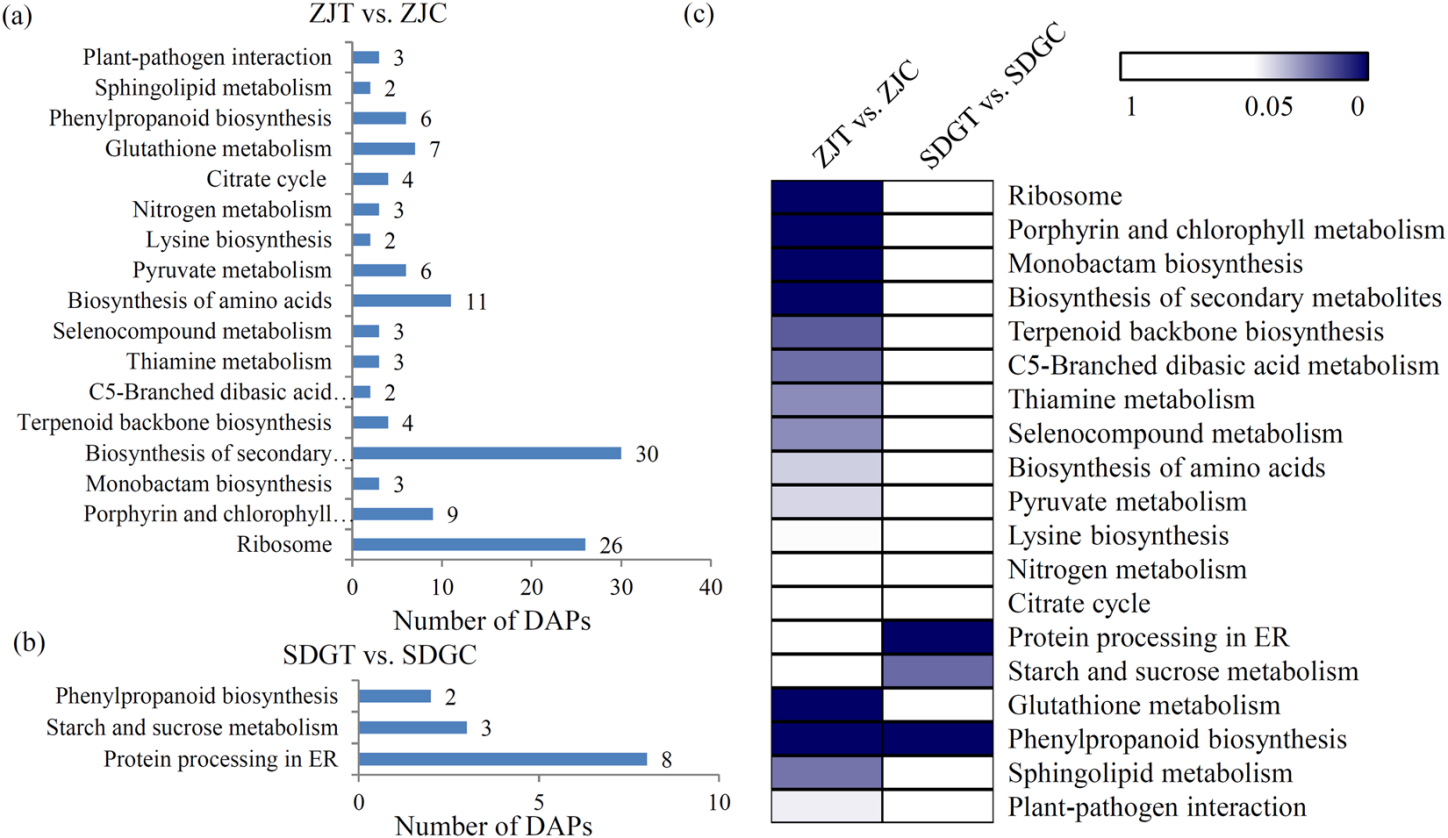
KEGG enrichment analysis of the DAPs in different comparisons. (**a**) The number of DAPs belonging to each enriched KEGG term in the ZJT vs. ZJC comparison. the SDGT vs. SDGC comparison (**b**). (**c**) The significant *P* values of each KEGG term in two different comparisons were shown by a heatmap. Blue indicated significantly enriched KEGG terms.

In total, 124 DAPs in the ZJT vs. ZJC comparison were classified into 17 significantly enriched KEGG pathways (*P* < 0.05) (Fig. 4a). The largest number of DAPs were enriched in ‘biosynthesis of secondary metabolites’, ‘ribosome’ and ‘biosynthesis of amino acids’ (Table S6). In addition, only 13 DAPs in the SDGT vs. SDGC comparison were classified into three significantly enriched KEGG pathways, including ‘protein processing in ER’, ‘starch and sucrose metabolism’, and ‘phenylpropanoid biosynthesis’ (*P* < 0.05) (Fig. 4b and Table S7). Interestingly, three pathogen defense-related pathways, including ‘terpenoid backbone biosynthesis’ and ‘selenocompound metabolism’, and ‘plant-pathogen interaction’, were significantly enriched in the ZJT vs. ZJC comparison rather than in the SDGT vs. SDGC comparison (Fig. 4c).

### DAPs related to terpenoid backbone biosynthesis

**Figure 5:**
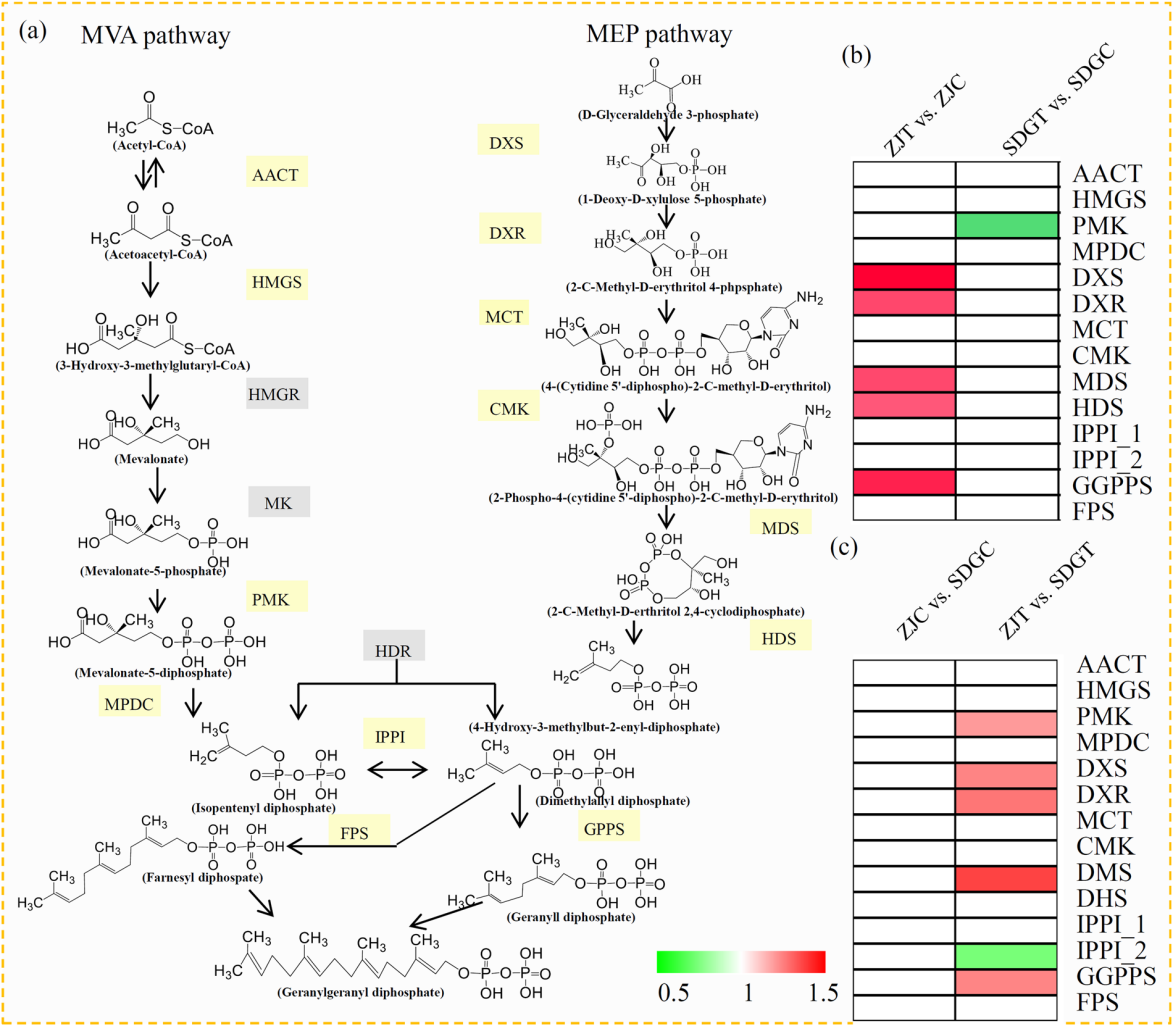
Analysis of the terpenoid backbone biosynthesis-related proteins under the *P*. *cubensis* infection. (**c**) Overview of the MVA and MEP pathways in cucumber. Yellow blocks indicated the identified proteins and grey blocks indicated undetected proteins. Enzyme abbreviations are: AACT: Acetyl-CoA C-acetyltransferase; HMGS: 3-Hydroxy-3-methylglutaryl-CoA synthase; HMGR: 3-Hydroxy-3-methylglutaryl-CoA reductase; MK: MVA kinase; PMK: Phospho-MVA kinase; MPDC: Diphospho-MVA decarboxylase; DXS: 1-Deoxy-D-xylulose 5-phosphate synthase; DXR: 1-Deoxy-D-xylulose 5-phosphate reductoisomerase; MCT: 2-C-methyl-D-erythritol 4-phosphate cytidylyltransferase; CMK: 4-(Cytidine 5-diphospho)-2-C-methyl-D-erythritol kinase; MDS: 2-C-methyl-D-erythritol 2,4-cyclodiphosphate synthase; HDS: 4-Hydroxy-3-methylbut-2-enyl-diphosphate synthase; HDR: 4-Hydroxy-3-methylbut-2-enyl diphosphate reductase; IPPI: Isopentenyl diphosphate-isomerase; GPPS: Geranyl diphosphate synthase; GGPPS: Geranylgeranyl diphosphate synthase. (**b**) Relative accumulation levels of proteins related to terpenoid backbone biosynthesis in the ZJT vs. ZJC and SDGT vs. SDGC comparisons. (**c**) Relative accumulation levels of proteins related to terpenoid backbone biosynthesis in the ZJC vs. SDGC and ZJT vs. SDGT comparisons. Red indicated up-regulated proteins and green indicated down-regulated proteins.

KEGG enrichment analysis showed that the ‘terpenoid backbone biosynthesis’ pathway was significantly enriched in the ZJT vs. ZJC comparison rather than in the SDGT *vs*. SDGC comparison. Our study identified most of the proteins participated in the essential steps of methylerythritol 4-phosphate (MEP) and mevalonate (MVA) pathways, which provide the precursors for terpenoid backbone biosynthesis^24^. In total, six key enzymes in the MEP pathway and our key enzymes in the MVA pathway were identified (Fig. 5a).

In the ZJ seedlings, the accumulation levels of DXS, DXR, MDS, HDS and GGPPS were up-regulated by the *P*. *cubensis* infection. In the SDG seedlings, only PMK was down-regulated by the *P*. *cubensis* infection (Fig. 5b). Under the control condition, no significantly changes in the accumulation levels of proteins related to terpenoid backbone biosynthesis were observed. After the *P*. *cubensis* infection, five enzymes, including PMK, DXS, DXR, DMK, and GGPPS, were significantly accumulated in ZJ than in SDG, and only IPPI_2 was significantly accumulated in SDG than in ZJ (Fig. 5c).

### Differential accumulation of pathogenesis-related proteins

Our data annotated a number of pathogenesis-related proteins, among which three endochitinases, one endoglucanase, seven peroxidases, one pathogenesis-related protein P2, and one lipid transfer protein were identified as DAPs under the *P*. *cubensis* infection (Table 1). The protein levels have been analyzed to reveal the effects of *P*. *cubensis* infection on these pathogenesis-related proteins. Under the *P*. *cubensis* infection, three endochitinases were up-regulated in the ZJ seedlings and only two endochitinases were up-regulated in the SDG seedlings. The accumulation level of endoglucanase was reduced in the ZJ seedlings and no significantly change was observed in the SDG seedlings. For peroxidases, all of them were induced in the ZJ seedlings and only two were induced in the SDG seedlings under the *P*. *cubensis* infection. Pathogenesis-related P2 protein was up-regulated in both of the ZJ and SDG seedlings under the *P*. *cubensis* infection. Accumulation level of lipid transfer protein 1 was reduced in the ZJ seedlings and no significantly change was observed in the SDG seedlings.

**Table 1:** Differential accumulation of pathogenesis-related proteins under the *P*. *cubensis* infection.

Protein accession	Protein description	ZJT/ZJC Ratio	ZJT/ZJC *P* value	SDGT/SDGC Ratio	SDGT/SDGC *P* value	Subcellular localization
Csa1G534750.1	Endochitinase	1.76	0.01	1.38	0.14	chloroplast
Csa6G509040.1	Endochitinase	1.46	0.01	1.03	0.76	chloroplast
Csa5G139760.1	Endochitinase	3.06	0.00	1.74	0.00	chloroplast
Csa4G051530.1	Endoglucanase	0.75	0.00	0.90	0.08	chloroplast
Csa6G495000.1	Peroxidase 73	1.90	0.00	1.54	0.00	chloroplast
Csa4G285780.1	Peroxidase 53	1.27	0.03	1.10	0.24	chloroplast
Csa4G285770.1	Peroxidase 2	1.37	0.00	1.10	0.04	extracellular
Csa4G285760.1	Peroxidase 2	1.23	0.05	1.04	0.70	chloroplast
Csa7G061710.1	Peroxidase N1	1.79	0.00	1.29	0.09	chloroplast
Csa4G285740.1	Peroxidase 2	1.39	0.00	0.98	0.78	vacuolar membrane
Csa3G736970.1	Peroxidase 21	1.40	0.02	1.24	0.00	chloroplast
Csa2G010390.1	Pathogenesis-related P2	2.22	0.03	1.35	0.04	extracellular
Csa1G228960.1	lipid transfer protein 1	0.59	0.04	0.99	0.97	chloroplast

A large number of HSPs were identified by the proteomes. In total, three HSPs were significantly induced and three HSPs were significantly reduced in the ZJ seedlings under the *P*. *cubensis* infection. In the SDG seedlings, 11 HSPs were significantly induced and only one HSP was reduced under the *P*. *cubensis* infection (Table 2).

**Table 2:** Differential accumulation of heat shock proteins.

Protein accession	Protein description	ZJT/ZJC Ratio	ZJT/ZJC *P* value	SDGT/SDGC Ratio	SDGT/SDGC *P* value	Subcellular localization
Csa3G183950.1	Heat shock protein 83	1.31	0.00	1.60	0.00	cytoplasm
Csa3G133380.1	Heat shock protein 26	0.77	0.00	0.84	0.00	cytoplasm
Csa1G024860.1	Heat shock protein 26 A	0.67	0.00	0.61	0.00	cytoplasm
Csa3G147740.1	Heat shock 701	1.01	0.94	1.42	0.05	cytoplasm
Csa3G113300.1	22.7 kDa heat shock protein	1.03	0.85	1.55	0.04	chloroplast
Csa5G591720.1	Heat shock protein 21	1.55	0.00	1.76	0.00	chloroplast
Csa7G312930.1	70 kDa heat shock-related protein	1.17	0.15	1.69	0.00	chloroplast
Csa5G190550.1	18.2 kDa heat shock protein	1.21	0.34	1.65	0.00	cytoplasm
Csa5G138480.1	Small heat shock protein 22	1.29	0.48	3.09	0.05	chloroplast
Csa2G070310.1	Heat shock 70 kDa protein	1.11	0.04	1.94	0.00	cytoplasm
Csa3G081340.1	21.7 kDa heat shock protein	0.71	0.00	1.65	0.00	nucleus
Csa5G135340.1	Heat shock protein 90-5	1.40	0.00	0.96	0.00	chloroplast
Csa4G646340.1	17.9 kDa heat shock protein	1.30	0.29	2.20	0.00	cytoplasm
Csa3G020080.1	Small heat shock protein 23	1.10	0.40	2.21	0.01	chloroplast

### Verification of several DAPs using PRM

For the identified DAPs, their antibodies are unavailable. To validate the differential accumulation of several key proteins involved in DM resistance, PRM was performed. In total, six key proteins, including endochitinase (Csa1G5347650.1), peroxidase 2 (Csa4G285770.1), HSP26A (Csa1G024860.1), HSP21 (Csa5G591720.1), PMK (Csa3G895920.1), and DMS 1 (Csa4G049620.), were selected for PRM verification. The relative abundances of several key proteins from different sample groups are presented in Fig. S2. The trend of these key proteins determined by PRM was consistent with our TMT-label quantification results.

## Discussion

DM is a major cucurbit foliar disease that defoliates cucumber crops and results to great economic losses^25^. Increasing works on the mechanisms of pathogen resistance explored many defense-related genes and proteins that might play a role in the DM resistance^26^. Therefore, more and more genetic resources, including potential genes and proteins associated with the DM resistance, must be explored to enhance the genetic improvement and resistance breeding of cucumber.

Previous proteomic analyses of cucumber have been performed using traditional methods, such as 2-D, which could detect a limited number of proteins^27^. For example, 71 differential protein spots were confidently identified by Du *et al*.^16^, 28 *Trichoderma asperellum* induced proteins were identified in cucumber root colonization by Segarra *et al*.^28^, 21 protein spots in Cd-treated cucumber were identified by Sun *et al*.^29^, 33 salt stress responsive protein spots were successfully identified by *Shao et al*.^30^, 63 DAPs in NaCl treated roots of cucumber were identified by Yuan *et al*.^16^, and 22 up- and 12 down-regulated proteins in cucumber roots under hypoxic stress were identified by Li *et al*.^31^. In our study, 6400 proteins were identified, which was far more than the protein numbers in the previous studies. A large number of identified proteins gave us an opportunity to reach a deeper analysis of proteins responsive to the *P*. *cubensis* infection.

Recently, transcriptomes of cucumber have identified a number of genes involved in response to the *P*. *cubensi*s infection. In Li’s study, 58 unique ESTs belonging to several categories involved in plant defense, such as signal transduction, cell defense, cell cycle, protein binding and metabolism, were identified^5^. In our study, DAPs involving “defense response” and “protein binding” were identified in SDG, suggesting that the genes belonging to above two categories were controlled at both the transcriptional and posttranscriptional levels. In Adhikari’s study, a number of defense related genes, including catalases, chitinases, lipoxygenases, and peroxidases, were rapidly induced by *P*. *cubensis* infection^20^. In our study, more defense related protein showed differential accumulation in ZJ (2 chitinases, 1 lipoxygenase and 5 peroxidases) than in SDG (1 chitinase and 1 peroxidase) (Table 1**)**, suggesting they were also controlled at both the transcriptional and posttranscriptional levels.

Comparative proteomic analysis identified 235 DAPs in the DM-resistant variety ZJ and 53 DAPs in the DM-susceptible variety SDG under the *P*. *cubensis* infection. Interestingly, at the early stage of *P*. *cubensis* infection, the number of differential expressed genes in the resistant line ‘PI 197088’ (4864 genes) was larger than that in the susceptible line ‘Vlaspik’ (1969 genes)^21^. The number of DAPs in the ZJ was larger than that in SDG, suggesting that a large-scale changes in proteomes occurred in resistant variety than susceptible variety.

The Ribosome-related proteins should be involved in the translational control in plants^32^. Glutathione-related proteins are known to be involved in redox control in response to a variety of stresses including pathogen infection^33^. For example, several glutathione S-transferase genes accumulated in the *Botryosphaeria dothidea*-infected Populus and were potentially related to pathogen resistance^34^. Interestingly, In our study, a number of DAPs in the ZJT vs. ZJC comparison were enriched in the “Ribosome” and “Glutathione metabolism” pathways, suggesting that translational control and redox control played an important role in the resistance against *P*. *cubensis* infection.

Terpenoids, the most diverse class of chemicals produced by plants, are involved in the protection in various abiotic environments, particularly in pathogenic fungi infection^35^. For example, terpenoids were predicted to be important defense metabolites in *Eucalyptus froggattii* seedlings^36^. Arbuscular mycorrhizal fungi-plant interaction induces the accumulation of terpenoids in plants^37^. Diterpenoid core is derived by isoprenoid precursor isopentenyl diphosphate (IPP) and dimethylallyl diphosphate (DMAPP), which are the downstream metabolites of the MEP and MVA pathways^24^. Under the *P*. *cubensis* infection, five key enzymes, including DXS, DXR, MDS, HDS, and GGPPS, were significantly up-regulated in ZJ, indicating an accumulation of terpenoids the DM-resistance variety ZJ. In Norway spruce, three DXS enzymes were differentially expressed under MeJA treatment, suggesting an important role of DXS in the induced defense response^38^. Over-expression of a GGPPS in white spruce saplings enhanced the accumulation of terpenoids and played a role in plant defense^39^. In cucumber, pathogen-induced terpenoids accumulation might play an important role in the resistance against *P*. *cubensis* infection.

A number of pathogenesis-related proteins, such as glucanases, chitinases, and peroxidases, were reported to be involved in plant defense^40^. Endo-glucanases, which accelerate the degradation of fungal cell wall, are induced by pathogen infection^41^. Endo-chitinases, another important class of the PR family, play a role in the hydrolysis of the major component of fungal cell wall, chitin^42^. In cucumber, three identified endochitinases were significantly up-regulated in ZJ and only one was significantly up-regulated in SDG. More glucanases were induced in the resistant cultivated variety than in the susceptible cultivated variety, suggesting that degradation of fungal cell walls might play a role in the resistance to pathogen infection^43^. Furthermore, uncontrolled reactive oxygen species (ROS) accumulation occurs under pathogen infection and results to the susceptibility of plants to fungal pathogens^44^. Peroxidases play an essential role in scavenging ROS in plant cells under pathogen infections^45^. In our study, all identified peroxidases were siginificantly up-regulated in ZJ and only two peroxidases were significantly up-regulated in SDG. Accumulation of peroxidases might contribute to the DM resistance of ZJ.

HSPs are quickly up-regulated by heat stress, and increasing studies showed a close relationship between HSPs and disease resistance^46^. For example, HSP90 plays a role in regulating the structure and stability of R proteins in plant defense responses^47^. HSP90 associated chaperoning is essential for the defense responses to rice blast fungus^48^. In addition to HSP90, HSP70 is also essential for plant resistance to pathogen infections^49^. HSP70 was involved in the resistance to *P*. *chicorii* infection in tobacco and resistance to oomycete pathogens in *Arabidopsis*^50^. In our study, a number of HSPs were identified in both two cucumber varieties. Most of the identified HSPs were significantly up-regulated by the *P*. *cubensis* infection in SDG rather than in ZJ, suggesting strong communications between heat shock responses and susceptibility to DM disease.

In conclusion, proteomes of DM resistant variety ZJ and DM susceptible variety SDG under the control and *P*. *cubensis* infection conditions were obtained. In total, 6400 proteins were identified using a MS/MS-based integrating TMT analysis strategy. Furthermore, 236 DAPs and 53 DAPs were identified in ZJ and SDG under the *P*. *cubensis* infection, respectively. A number of terpenoid backbone synthesis enzymes, pathogenesis-related proteins, and HSPs showed significantly differences between ZJ and SDG under the *P*. *cubensis* infection, suggesting that DM resistance was conducted by a complex network.

## Supplementary information

Supplementary Information

Table S1-S7
