# Supplementary material for: Comparative proteomic analysis provides insights into the complex responses to Pseudoperonospora cubensis infection of cucumber (Cucumis sativus L.)
Source: Sci Rep. 2019 Jul 1;9:9433. doi: 10.1038/s41598-019-45111-4 (PMC6603182; doi:10.1038/s41598-019-45111-4)
Supplement: Supplementary file 1 — Supplementary Information [file 41598_2019_45111_MOESM1_ESM.docx]

**Comparative proteomic analysis provides insights into the complex responses to *Pseudoperonospora cubensis* infection of cucumber (*Cucumis sativus* L.)**

Peng Zhang^1^, Yuqiang Zhu^1^, Xiujun Luo ^2^ and Shengjun Zhou^1*^

^1^ Institute of Vegetable, Zhejiang Academy of Agriculture Sciences, Hangzhou, China

^2^College of Life and Environmental Science, Hangzhou Normal University, Hangzhou 310036, China;

*Corresponding author:

Shengjun Zhou, Email: yinxiang0586@sohu.com


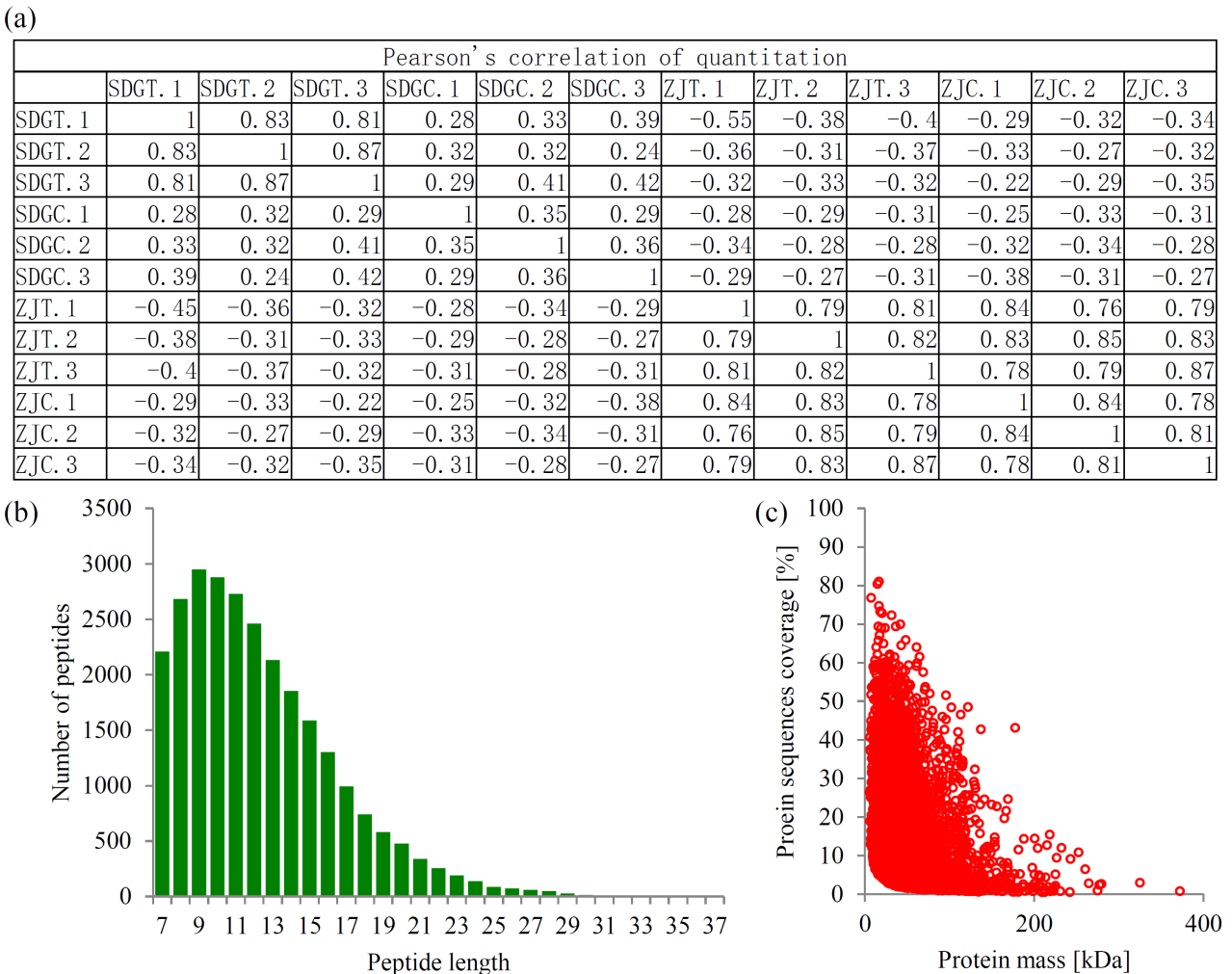


Fig. S1 **Quantitative proteome analysis and QC validation of MS data.** Protein from each fruit group were extracted in three biological replicates. Proteins were trypsin digested and then analyzed by HPLC-MS/MS. (a) Pearson’s correlation of proteomes from different sample groups. (b) Length distribution of all identified peptides. (c) Mass delta of all identified peptides.


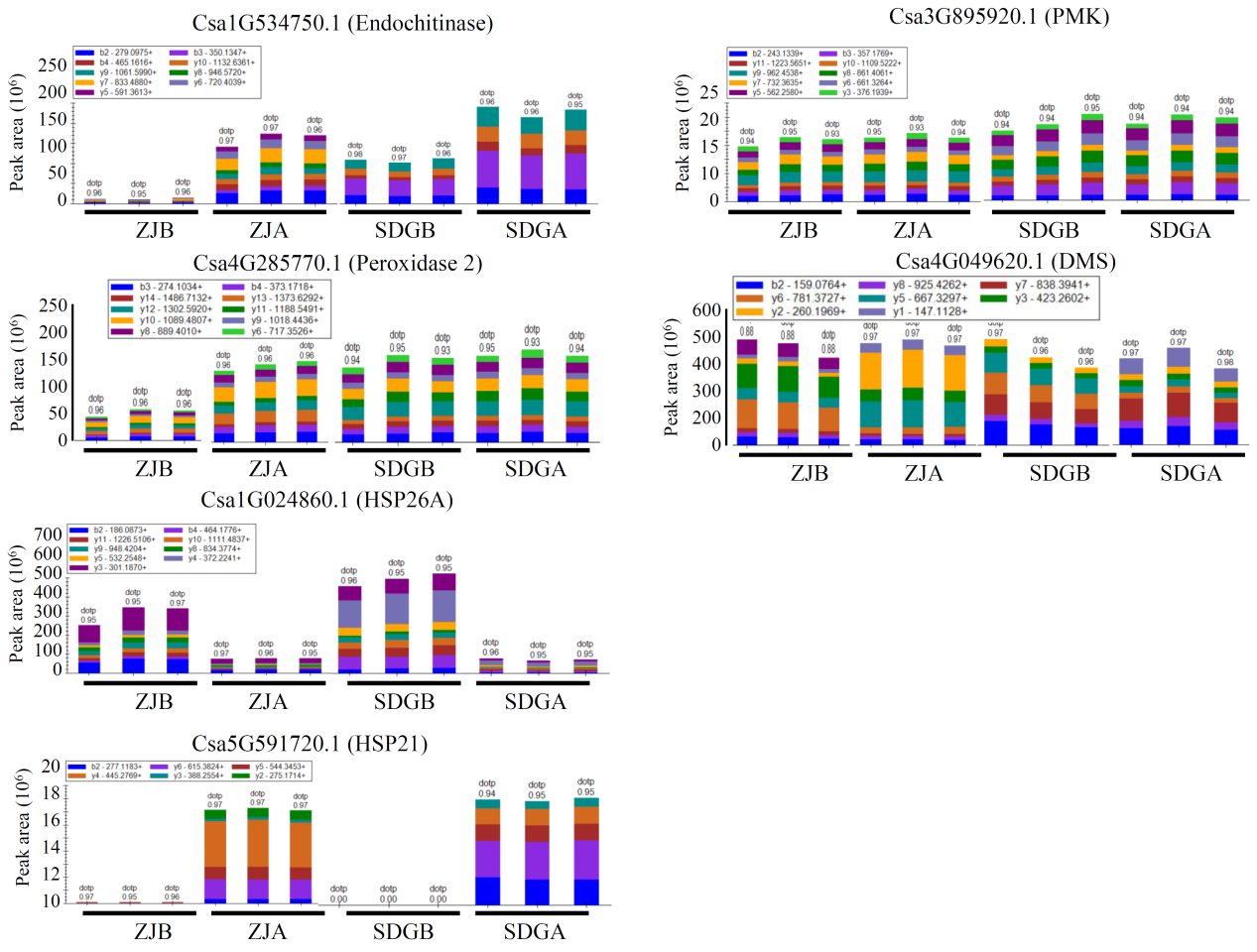
Figure S2 **Verification of several DAPs using PRM.** Six representative proteins, including two pathogenesis-related proteins, two HSPs, and two terpenoid backbone biosynthesis-related protein, were randomly selected for PRM verification.
